# Endoplasmic reticulum stress caused by traumatic injury promotes cardiomyocyte apoptosis through acetylation modification of GRP78: GRP78 acetylation modification and cardiomyocyte apoptosis

**DOI:** 10.3724/abbs.2023277

**Published:** 2023-12-15

**Authors:** Zi Yan, Yufeng Liu, Bowen Yang, Wenhui Zhao, Yan Wang, Deping Wang, Jianguo Li, Xiangying Jiao, Jimin Cao

**Affiliations:** 1 Department of Physiology Shanxi Medical University Taiyuan 030001 China; 2 State Key Laboratory of Cellular Physiology Shanxi Medical University Taiyuan 030001 China; 3 the First Clinical Medical College Shanxi Medical University Taiyuan 030001 China; 4 Guangdong Province Key Laboratory of Psychiatric Disorders Guangzhou 510515 China

**Keywords:** acetylation, apoptosis, cardiomyocytes, GRP78, trauma

## Abstract

Cardiomyocyte apoptosis is an important cause of trauma-induced secondary cardiac injury (TISCI), in which the endoplasmic reticulum stress (ERS)-mediated apoptosis signaling pathway is known to be first activated, but the mechanism remains unclear. In this study, rat models of traumatic injury are established by using the Noble-Collip trauma device. The expression of glucose-regulating protein 78 (GRP78, a molecular chaperone of the cardiomyocyte ER), acetylation modification of GRP78 and apoptosis of cardiomyocytes are determined. The results show that ERS-induced GRP78 elevation does not induce cardiomyocyte apoptosis in the early stage of trauma. However, with prolonged ERS, the GRP78 acetylation level is elevated, and the apoptosis of cardiomyocytes also increases significantly. In addition, in the early stage of trauma, the expression of histone acetyl-transferase (HAT) P300 is increased and that of histone deacetylase 6 (HDAC6) is decreased in cardiomyocytes. Inhibition of HDAC function could induce the apoptosis of traumatic cardiomyocytes by increasing the acetylation level of GRP78. Our present study demonstrates for the first time that post-traumatic protracted ERS can promote cardiomyocyte apoptosis by increasing the acetylation level of GRP78, which may provide an experimental basis for seeking early molecular events of TISCI.

## Introduction

With socioeconomic development, the number of traumatic patients from traffic and industrial accidents has been gradually increasing annually, among which cardiac damage is a predictive factor of post-traumatic adverse prognosis. Clinical studies have demonstrated that trauma can not only cause direct injuries to the heart, such as arrhythmia, abnormal ventricular wall motion, myocardial wall rupture, valvular injury, and myocardial contusion [1], but also trigger trauma-induced secondary cardiac injury (TISCI) [2], presenting as cardiac dysfunction several days or weeks after trauma, although there is no abnormality in cardiac function indexes within 24 h after trauma [3]. The onset of TISCI is insidious and likely to be misdiagnosed, causing a high risk of mortality. It is one of the main causes of death among young and middle-aged people worldwide [ 4, 5]. However, the exact mechanism of TISCI remains unclear.

Research has demonstrated that cardiomyocyte apoptosis is the main cause of TISCI [6]. Endoplasmic reticulum stress (ERS) participates in the development and progression of cardiovascular diseases [7]. It has been reported that among the three major caspase pathways causing cardiomyocyte apoptosis in traumatic rats, the ERS apoptosis pathway is the earliest to be activated [8]. As an ER molecular chaperone, the expression of glucose regulating protein 78 (GRP78) is elevated during the process of ERS and participates in the unfolded protein response. The increased expression of GRP78 can protect cells by maintaining ER homeostasis through inducing an adaptive response [9]. Numerous studies have demonstrated that GRP78 can undergo multiple posttranslational modifications (PTMs), such as acetylated, sulfenylated, glutathionylated, ADP-ribosylated, phosphorylated, AMPylated, and citrullinated modifications [10]. Acetylation is a common PTM under the regulation of histone acetyl-transferase (HAT) and histone deacetylase (HDAC). Some studies have noted that GRP78 acetylation can induce apoptosis in breast cancer cells [ 11, 12]. However, how the expression of GRP78 in cardiomyocytes undergoes a change in the early phase of trauma and whether GRP78 participates in the apoptosis of traumatic cardiomyocytes through self-acetylation remain unclear.

In the present study, we established a rat model of Noble-Collip drum trauma to investigate the expression of GRP78 and its acetylation regulatory mechanism for the sake of exploring the underlying mechanisms of GRP78 in trauma-induced cardiomyocyte apoptosis.

## Materials and Methods

### Establishment of the rat trauma model

Forty-eight male rats aged 6‒8 weeks and weighing 180‒200 g (Animal Center of Shanxi Medical University, Taiyuan, China) were equally randomized to a sham-trauma group (sham group) and 5 trauma groups at 0, 3, 6, 12 and 24 h post-trauma. Using the same method of establishing the rat trauma model in our prior studies [ 8, 13], the rats were anesthetized by intraperitoneal injection and then placed in a 30-cm diameter Noble-Collip trauma device (WYM-1; Thomas Jefferson University, Philadelphia, USA), which ran 5 min at a 40 rpm for 200 resolutions. Rats in the sham group were fixed with tape in the trauma device without being injured by falling during the process of rotation. Rats in the 5 trauma groups were injured by each fall during rotation.

### Cell culture and treatment with Trichostatin A or ITSA-1

Using the preliminary experimental method [14], H9C2 cardiomyocytes (Cas9X, Suzhou, China) were cultured in Dulbecco’s modified Eagle’s medium (DMEM; Boster, Wuhan, China) containing 10% fetal bovine serum (FBS, SA211; CellMax, Beijing, China), which was used as the normal plasma group (NP group). H9C2 cardiomyocytes were cultured with medium containing 10% rat serum collected at 12 h post-trauma for 6, 12 and 24 h as the trauma groups (TP group). Cardiomyocytes in the trauma groups were cultured with 0.5 μM trichostatin A (HY-15144; MedChemExpress, Monmouth Junction, USA) and 10 μM ITSA-1 (HY-100508; MedChemExpress) for 24 h.

### Western blot analysis

The protein expression levels of GRP78, P300, HDAC6, caspase3 and cleaved caspase3 were detected by western blot analysis. Rat myocardial tissue or cardiomyocytes were lysed on ice using pre-cooled RIPA lysis buffer (AR0102; Boster) for 1 h and centrifuged at 13,800 *g* for 10 min at 4°C to collect the supernatant protein. After measuring the protein concentration using a BCA Protein Assay Kit (Boster), the sample was boiled in a 100°C bath for 10 min and separated by SDS-PAGE after completely cooling to room temperature. After electrophoresis, the sample was transferred to a PVDF membrane (Solarbio, Beijing, China). The membrane was blocked with 5% skim milk for 2 h and cultured with primary antibodies against GRP78 (ab21685; Abcam, Cambridge, UK), HDAC6 (ab1440; Abcam), P300 (57625; Cell Signaling, Boston, USA), caspase3 (ab179517; Abcam), β-actin (AP0060; Bioworld Technology Co., Ltd), and GAPDH (60004-1-Ig; Proteintech, Chicago, USA) at 4°C overnight, followed by incubation with secondary antibodies (BA1054; Boster) for 2 h at room temperature. The ECL Plus Western Blotting Substrate (AR1196-200; Boster) was spread flat on the PVDF membrane, and protein bands were visualized with a UVP BioSpectrum 810 imaging system. Protein analysis was performed using ImageJ Software, and the gray value ratio of the target protein and β-actin or GAPDH was used as the protein expression level.

### Quantitative real-time PCR (RT-qPCR)

The mRNA expression levels of GRP78, HDAC6 and P300 in cardiomyocytes were detected by RT-qPCR. Total RNA was extracted by adding 50 g rat myocardial tissue or 5×10 ^6^ H9C2 cardiomyocytes in 1 mL TRIgent (MF034; Mei5bio, Beijing, China). After measuring the mRNA concentration, the RNA was reverse transcribed using the M5 Sprint qPCR RT kit with gDAN removed (MF949; Mei5bio) to obtain cDNA. Finally, amplification was performed using 2×M5 Hiper SYBR Premix EsTaq (with Tli RnaseH) (MF787; Mei5bio) to determine the corresponding mRNA level. The primers used are listed in Table 1.

**Table:** **Table 1** Sequences of primers used for RT-qPCR

Primer	Sequence (5′→3′)
*GRP78* forward	CTTGGTATTGAAACTGTGGGAG
*GRP78* reverse	CCTTGATTGTTACGGTGGGCTG
*HDAC6* forward	ACCATGCTGCCTGTTTACCTTCTG
*HDAC6* reverse	CACCACTGCCACTTGTCTCCTTC
*P300* forward	ACCTTCTCCTGTTCCTAGCCGTAC
*P300* reverse	AATTGCTGTTGCTGCTGGTTGTTG
*GAPDH* forward	GGCACAGTCAAGGCTGAGAATG
*GAPDH* reverse	ATGGTGGTGAAGACGCCAGTA

### Immunoprecipitation (IP) assay

The acetylation level of GRP78 was detected using a Pierce® Crosslink IP Kit (26147; Thermo Fisher Scientific, Waltham, USA). Precooled IP lysis/wash buffer was added to the rat myocardial tissue or H9C2 cells to obtain the cell lysate. Protein A/G agarose beads and anti-GRP78 antibody (11587-1-AP; Proteintech) were added to Pierce Spin Columns to enable binding. Then, the DSS cross-linked antibody was added to Pierce Spin Columns. The well-prepared cell lysate was added to Pierce Spin Columns containing the antibody-crosslinked resin and cultured at 4°C overnight. On the following day, the final protein product was harvested using elution buffer. The collected protein sample was added to loading buffer, heated at 100°C for 5 min, cooled to room temperature, and finally analyzed by SDS-PAGE.

### Immunohistochemistry (IHC) staining

Rat myocardial tissue blocks were fixed in 4% formalin, paraffin-embedded, and sliced into 4-μm sections. After baking, the sections were dewaxed and hydrated using xylene and anhydrous ethanol at different concentrations. The antigen was repaired by sodium citrate buffer solution, cooled to room temperature, and H _2_O _2_ (SV0002; Boster) was added. After eliminating endogenous peroxidase activity, the sample was blocked with BSA. Primary antibodies against GRP78 (ab21685; Abcam), HDAC6 (ab1440; Abcam) and P300 (ab275379; Abcam) were added by dripping and incubated in a humid environment at 37°C for 2 h or 4°C overnight. On the following day, the polymeric horseradish peroxidase (HRP)-labeled antibody was added by dripping, followed by incubation in a humid environment at 37°C for 30 min, and finally visualized using a DAB kit (AR1027; Boster).

### TUNEL assay

Early-stage apoptosis in the myocardial tissue was detected using an *In Situ* Cell Death Detection Kit, POD (11684817910; Wolcavi, Beijing, China). The basic staining procedures were as follows: rat myocardial tissue was fixed, embedded, sliced, dewaxed with gradient alcohol, hydrated with xylene, treated with proteinase K for 15‒30 min, and washed with PBS twice. The prepared TUNEL mixed solution (deoxyribonucleotide terminal transferase+fluorescein-labeled dUT) was dripped onto the tissue, incubated in a wet box for 1 h at 37°C, washed with PBS 3 times, dripped with Converter POD at 37°C, incubated in a wet box for 30 min, washed with PBS 3 times, dripped with DAB, incubated at room temperature for 10 min, rinsed, re-stained with hematoxylin, dehydrated with gradient alcohol, hyalinized with xylene, and sealed with neutral resin. Apoptotic cells were observed and photographed under an optical microscope (Nikon, Tokyo, Japan).

### Cell cycle and apoptosis analysis

Cell cycle progression and apoptosis were detected using an Annexin V-PI cell apoptosis assay kit (C0007-100; APPLYGEN, Beijing, China). H9C2 cells were collected with the cell culture solution preserved, washed with PBS twice, supplemented with an appropriate amount of trypsin without EDTA according to the number of cells added, and digested in the cultivator for approximately 1 min. The above preserved culture solution was added to terminate the digestion. The cell suspension was collected in a 10 mL centrifuge tube, centrifuged at 100‒400 *g* for 5 min, washed with precooled PBS twice, and 100 μL of binding buffer was added to resuspend the cells. The resuspended cells were mixed with 5 μL Annexin V-FITC, incubated for 15 min away from light, and mixed again with 10 μL PI solution 5 min before detection. After being resuspended in 400 μL PBS, the cells were analyzed by flow cytometry.

### Immunofluorescence (IF) staining

H9C2 cells growing on the glass slide were fixed with 4% polyformaldehyde at room temperature for 20 min, washed with precooled PBS 3 times for 5 min, added to 0.01% Triton X-100, well ventilated at room temperature for 10 min, washed with PBS 3 times for 5 min, sealed with PBST containing 1% BSA at room temperature for 1 h, and incubated with 500 μL diluted antibodies GRP78 (ab21685; Abcam) and caspase3 (ab179517; Abcam) at 4°C overnight. On the following day, the cells were washed with PBST 3 times for 5 min, incubated with 1:200 diluted HRP-labelled antibody in a wet box for 1 h, and washed with PBST 3 times for 5 min. After adding a drop of DAPI (AR1176; Boster) to the center of the glass slide, the cells were incubated for 5‒10 min away from light and washed with PBST 3 times for 5 min. After dripping a drop of anti-fluorescence attenuation sealing agent (AR1109; Boster), images were captured under a fluorescence microscope (Nikon).

### Statistical analysis

Data were analyzed using GraphPad Prism 8.3.0, and expressed as the mean±SEM. Comparisons between multiple groups were performed by one-way ANOVA and LSD- *t* test. *P*<0.05 was considered significantly different.

## Results

### ERS-induced early GRP78 expression during traumatic injury does not lead to cardiomyocyte apoptosis

**Figure 1:**
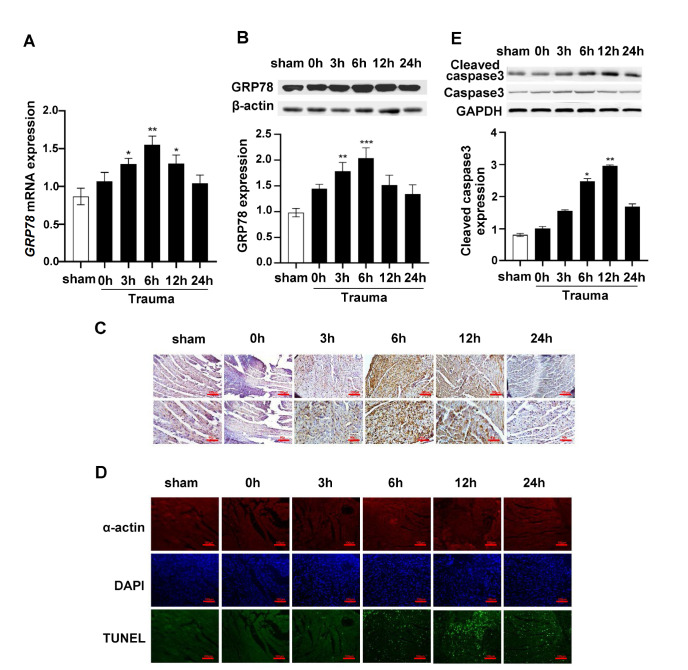
Figure 1 ERS-induced early GRP78 expression during traumatic injury does not cause cardiomyocyte apoptosis (A) RT-qPCR analysis of the mRNA expression of GRP78 in rat cardiomyocytes at different time points after trauma. (B) Representative western blots and quantification showing the protein expression of GRP78 in rat cardiomyocytes at different time points after trauma. (C) Representative IHC assay of GRP78 in rat cardiomyocytes at different time points after trauma. Brown particles indicate the positively stained cells. Scale bars: 100 μm (upper) and 50 μm (lower). (D) TUNEL staining. Cardiomyocytes were labeled with an anti-α-actin antibody (red), the nuclei were stained with DAPI (blue), and apoptotic nuclei were identified by TUNEL staining (green). Scale bar: 100 μm. (E) Representative western blots and quantification showing the protein expression of cleaved caspase3 in rat cardiomyocytes at different time points after trauma. Data are expressed as the mean±SEM (n=8 per group). *P<0.05, **P<0.01, ***P<0.001 vs the sham group.

GRP78 is a molecular chaperone that performs critical functions in the ER. In myocardial tissue, RT‒qPCR showed that *GRP78* mRNA expression was markedly increased 3 h after trauma and remained high 6 h and 12 h after trauma ( Figure 1A). The protein expression of GRP78 in the myocardial tissue began to increase 3 h after trauma and reached the peak at 6 h ( Figure 1B). IHC staining also showed that the strongest staining intensity occurred 3 h and 6 h after trauma ( Figure 1C). However, cardiomyocyte apoptosis increased significantly 6 h after trauma and reached a maximum 12 h after trauma ( Figure 1D,E). The above results suggested that the early elevation of the ERS marker GRP78 did not induce the occurrence of apoptosis in cardiomyocytes.

### Traumatic injury-induced apoptosis of cardiomyocytes correlates with GRP78 acetylation levels

**Figure 2:**
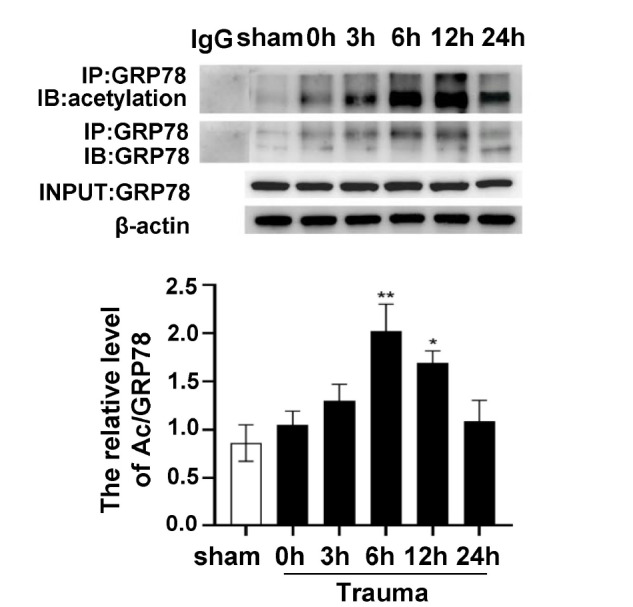
Figure 2 Time course of myocardial GRP78 acetylation determined by IP after trauma Rat cardiomyocytes were lysed, and GRP78 was immunoprecipitated from the lysates followed by immunoblotting with anti-acetylated-lysine antibody. Alternatively, the lysates were immunoblotted with GRP78 and β-actin. Data are expressed as the mean±SEM (n=8 per group). *P<0.05, **P<0.01 vs the sham group.

GRP78 is regulated by acetylations. The IP results showed that the level of GRP78 acetylation in the myocardial tissue was significantly elevated 6 h and 12 h after trauma ( Figure 2), which coincided with the significant increase in cardiomyocyte apoptosis levels at 6 h and 12 h post-trauma. These results suggested that post-traumatic ERS-induced apoptosis of cardiomyocytes was associated with GRP78 acetylation levels.

### There exists a dynamic balance between HAT and HDAC in myocardial tissue during trauma-induced ERS

**Figure 3:**
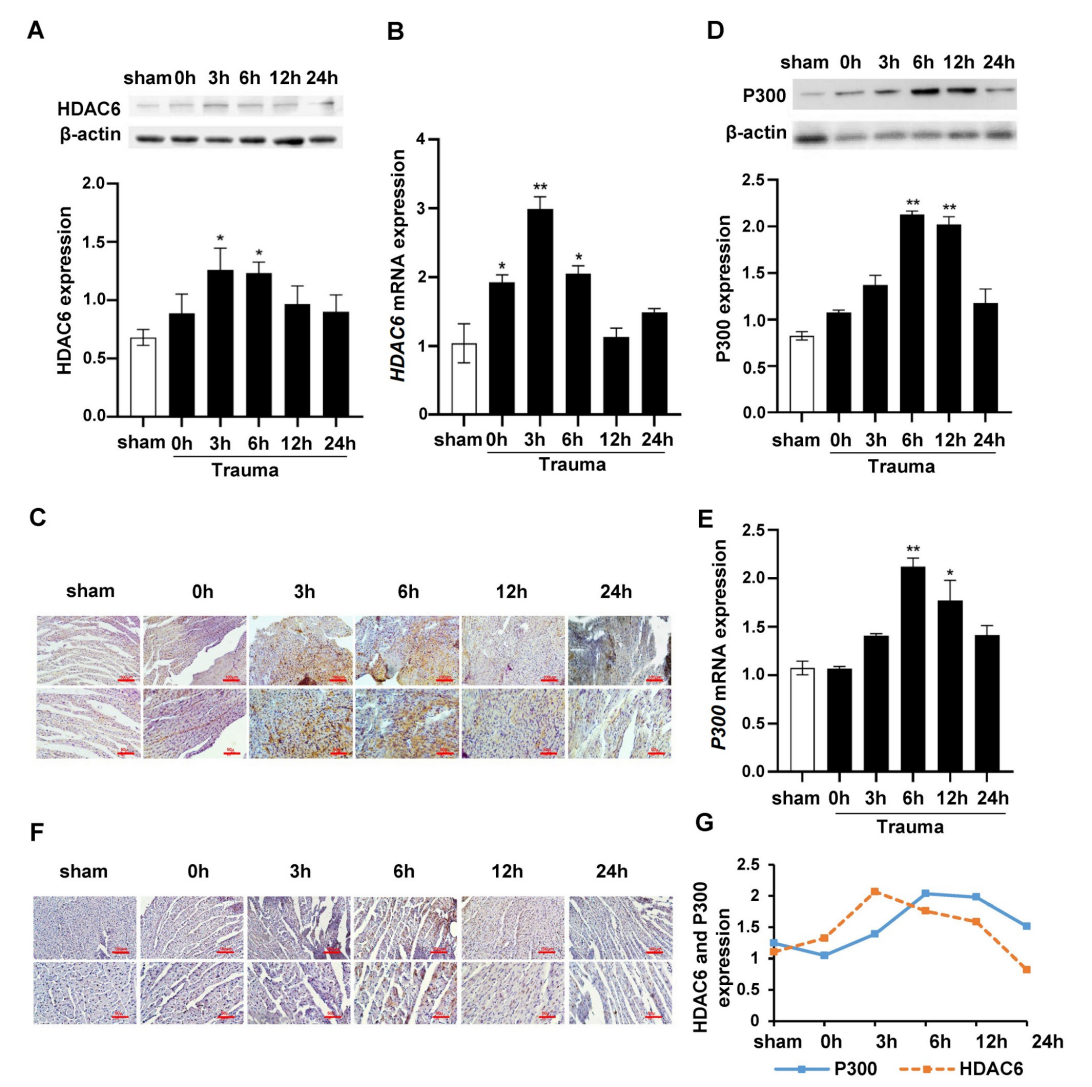
Figure 3 Dynamic equilibrium of HAT (P300) and HDAC (HDAC6) during ER stress (A) Representative western blots and quantification showing the protein expression of HDAC6 in rat cardiomyocytes at different time points after trauma. (B) RT‒qPCR analysis of the mRNA expression of HDAC6 in rat cardiomyocytes at different time points after trauma. (C) Representative IHC assay of HDAC6 in rat cardiomyocytes at different time points after trauma. Brown particles indicate positively stained cells. Scale bars: 100 μm (upper) and 50 μm (lower). (D) Representative western blots and quantification showing the protein expression of P300 in rat cardiomyocytes at different time points after trauma. (E) RT‒qPCR analysis of the mRNA expression of P300 in rat cardiomyocytes at different time points after trauma. (F) Representative IHC assay of P300 in rat cardiomyocytes at different time points after trauma. Brown particles indicate positively stained cells. Scale bars: 100 μm (upper) and 50 μm (lower). (G) Line graph of the expression trend for HDAC6 and P300. Data are expressed as the mean±SEM (n=8 per group). *P<0.05, **P<0.01 vs the sham group.

Studies have proven that P300 and HDAC6 molecules are regulators of the HAT and HDAC of GRP78, respectively [ 11, 12]. Our western blot analysis showed that the expression level of HDAC6 protein in the myocardial tissue peaked at 3 h after trauma ( Figure 3A), which is consistent with the IHC staining results ( Figure 3C). RT‒qPCR showed that the mRNA expression level of *HDAC6* in the myocardial tissue began to increase immediately after trauma and reached its maximum at 3 h ( Figure 3B). In addition, the expression of P300 protein in the myocardial tissue was markedly elevated 6 h and 12 h after trauma ( Figure 3D,F), and the expression of *P300* mRNA reached a maximal level at 6 h ( Figure 3E). Over time, the expressions of HDAC6 and P300 exhibited an ebb-and-flow trend ( Figure 3G). All the above results suggested that the trauma-induced elevation of GRP78 acetylation in myocardial tissue was correlated with increased expression of P300 and decreased expression of HDAC6.

### Inhibition of HDAC function promotes apoptosis of traumatic cardiomyocytes by increasing the acetylation level of GRP78

**Figure 4:**
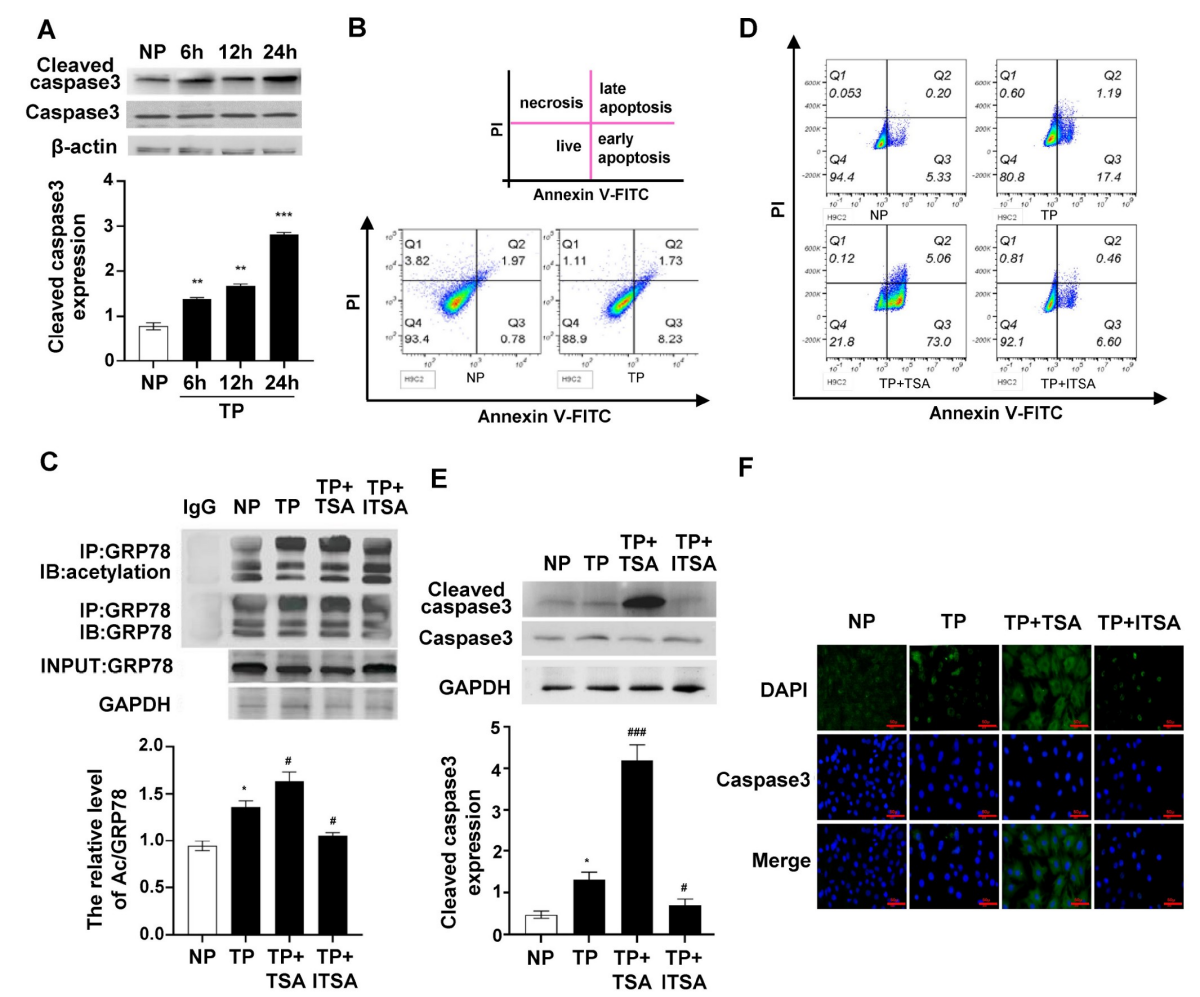
Figure 4 The apoptosis level is obviously increased after HDAC inhibition (A) Representative western blots and quantification showing the protein expression of cleaved caspase3 in H9C2 cells induced by traumatic serum at 6, 12 and 24 h. (B) Flow cytometric analysis of apoptosis of H9C2 cells 24 h after culture with traumatic serum by Annexin V/propidium iodide staining. (C) H9C2 cells were lysed after pretreatment with TSA or ITSA-1, and GRP78 was immunoprecipitated from the lysates, followed by immunoblotting with anti-acetylated-lysine antibody. Alternatively, the lysates were immunoblotted with GRP78 and GAPDH. (D) Flow cytometric analysis of apoptosis of H9C2 cells pretreated with TSA or ITSA-1 by Annexin V/propidium iodide staining. (E) Representative western blots and quantification showing the protein expression of cleaved caspase3 in H9C2 cells pretreated with TSA or ITSA-1. (F) Representative IF assay of caspase3 in H9C2 cells pretreated with TSA or ITSA-1. The cell nuclei were stained with DAPI (blue), and the caspase3 protein was detected by anti-caspase3 antibody with fluorescence (green) and merged images. Scale bar: 100 μm. Data are expressed as the mean±SEM (n=6 per group). *P<0.05, **P<0.01, ***P<0.001 vs NP group; #P<0.05, ###P<0.001 vs TP group.

To further explore the relationship between the acetylation level of GRP78 and cardiomyocyte apoptosis, we established an isolated myocardial injury model by culturing H9C2 cells in the serum of traumatic rats [15]. The results showed that cardiomyocyte apoptosis was most pronounced 24 h after trauma ( Figure 4A), and the number of apoptotic cells was increased markedly in the early stage ( Figure 4B). IP analysis showed that the acetylation level of GRP78 was increased markedly in the HDAC inhibitor trichostatin A (TSA) group and decreased obviously in the HDAC activator ITSA-1 group ( Figure 4C). Flow cytometry results showed that the number of early apoptotic cells accounted for approximately 73% of the total number of cells in the TSA group, while the number of apoptotic cells in the ITSA-1 group decreased to some extent compared with that in the trauma groups ( Figure 4D). Both western blot analysis and IF staining results showed that the expression level of cleaved caspase3 in the TSA group was significantly higher than that in the normal plasma group ( Figure 4E,F). Taken together, these results demonstrated that inhibition of HDAC function could increase the apoptosis of traumatic cardiomyocytes.

### GRP78 acetylation reduces its protein expression without affecting mRNA expression

**Figure 5:**
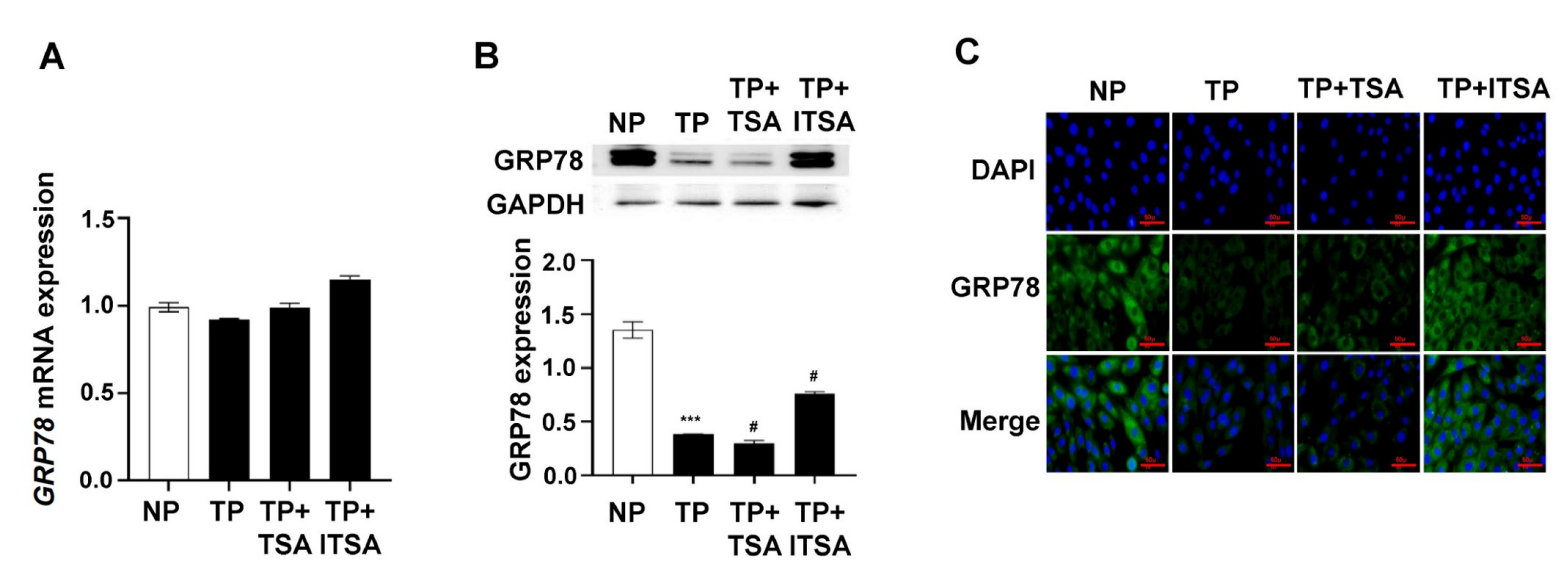
Figure 5 GRP78 acetylation reduces GRP78 translation but does not affect GRP78 transcription (A) RT-qPCR analysis of GRP78 mRNA expression in H9C2 cells pretreated with TSA or ITSA-1. (B) Representative western blots and quantification showing the protein expression of GRP78 in H9C2 cells pretreated with TSA or ITSA-1. (C) Representative IF assay of GRP78 in H9C2 cells pretreated with TSA or ITSA-1. The cell nuclei were stained with DAPI (blue), and the GRP78 protein was detected by anti-GRP78 antibody with fluorescence (green) and merged images. Scale bar: 100 μm. Data are expressed as the mean±SEM (n=6 per group). ***P<0.001 vs NP group; #P<0.05 vs TP group.

At the same time, by modifying GRP78 acetylation, HDAC can regulate the expression of functional proteins by modifying histones [16]. Therefore, we detected the change in GRP78 expression after inhibiting HDAC function via TSA. The RT‒qPCR results showed no significant change in the expression of *GRP78* mRNA ( Figure 5A), while western blot analysis showed that the protein expression of GRP78 was decreased markedly ( Figure 5B). IF staining showed that the number of positive cells was also significantly decreased ( Figure 5C).

## Discussion

In this study, we found that increased expression and acetylation of GRP78 in cardiomyocytes of traumatic rats were associated with cardiomyocyte apoptosis and that inhibition of HDAC function promoted the apoptosis of traumatic cardiomyocytes by increasing the acetylation level of GRP78.

TISCI refers to cardiac dysfunction occurring several days or several weeks after trauma, with a high risk of mortality. Cardiomyocyte apoptosis is the main reason for TISCI-induced cardiac dysfunction [ 6, 17]. Apoptosis is a basic biological phenomenon of cells that maintains a dynamic balance in the number of cells by removing unwanted or abnormal cells. Like neurons, cardiomyocytes are non-renewable. Once excessive loss of cardiomyocytes occurs, the body undergoes compensatory ventricular remodeling. As a result, the electrophysiological activity performed by cardiomyocytes becomes abnormal, and the atrial and ventricular systolic and diastolic activities are inhibited, ultimately affecting the pumping function of the heart. In the present study, cardiomyocyte apoptosis was determined by caspase3 expression detection and TUNEL staining. The results showed that cardiomyocytes exhibited different degrees of apoptosis at different time points after trauma, which proves that trauma could cause cardiomyocyte apoptosis.

There are three major pathways leading to apoptosis: the death receptor pathway, the mitochondrial pathway, and the ERS response pathway [18]. Our previous study demonstrated that caspase12, an ERS-related biological marker [19], was first elevated after trauma [8], indicating that the ERS response pathway was first activated. GRP78 is a member of the heat-shock protein 70 (HSP70) family. It is present on the ER of all eukaryotes as an ER molecular chaperone. In normal cells, GRP78 can bind to and inactivate the three ER stress transducers-activating transcription factor 6 (ATF6), protein kinase R-like endoplasmic reticulum kinase (PERK), and inositol-requiring enzyme 1α (IRE1α). When the body is subjected to stress responses such as nutrient deficiency, hypoxia, low pH, secretory protein mutation [20], excessive production of free radicals [21], or use of drugs [22], cells can produce large amounts of misfolded proteins. Under such conditions, GRP78 preferably binds with these misfolded proteins to induce dissociation activation of the three ER transducers. Activated PERK phosphorylates and activates the eukaryotic translation initiation factor eIF2α. Activated eIF2α can inhibit continuous protein synthesis and prevent the synthesis of unfolded or misfolded proteins in the early stage of ERS, thus facilitating the recovery of ER function. ATF6 enters the Golgi apparatus and is cleaved into active transcription factors. Then, it migrates to the nucleus to upregulate proteins that increase the folding capacity of the endoplasmic reticulum. IRE1α has endonuclease activity, which splices a 26-base intron from the mRNA encoding XBP1 and results in the translation of XBP1, accelerating the expressions of genes needed to restore endoplasmic reticulum function and protein-degrading genes. The activation of ATF6, PERK and IRE1α promotes the synthesis of GRP78 and other ER molecular chaperones to maintain ER homeostasis and simultaneously increases the expressions of apoptotic proteins, leading to the occurrence of apoptosis [ 12, 23, 24]. GRP78 participates in the unfolded protein response (UPR) due to the small patch of its hydrophobic structure. This hydrophobic structure can recognize unfolded or misfolded proteins, which leads to the refolding and assembly of unfolded or misfolded proteins and ER-associated protein degradation (ERAD) to reduce unfolded or misfolded proteins within the ER, thus restoring ER homeostasis [ 23‒ 25]. In breast cancer cells overexpressing GRP78, the expression level of anti-apoptotic protein Bcl-2 was increased, while the expression of pro-apoptotic proteins Bax and Bim was decreased. The function of GRP78 in promoting cell survival is represented by its anti-apoptosis feature, and therefore, it is recognized as a survival factor that has experienced ERS [26]. In addition, overexpression of GRP78 could increase the resistance of breast cancer to adriamycin [27]. In the present study, we found that the expression of GRP78 in myocardial tissue was increased markedly 3 h after trauma and persisted until 12 h after trauma, while the number of TUNEL-positive cells and the expression of cleaved caspase3 significantly increased 6 h after trauma. This result suggests that ERS-induced elevation of GRP78 in the early stage of trauma does not induce cardiomyocyte apoptosis. However, with prolonged ERS, apparent cardiomyocyte apoptosis was observed even though the expression of GRP78 remained at a high level, which is consistent with the finding that prolonged ERS induces cell apoptosis [9].

In addition to acetylating modifications in histones, non-histones are also regulated by acetylating modifications [28]. The acetylation level of GRP78 is regulated by HATs and HDACs. Protein acetylation is mainly catalyzed by five HATs (CBP, P300, GCN5, PCAF and TIP60), while more than two-thirds of the acetylation sites of lysine are the target points of CBP and/or P300. Both CBP and P300 have strong acetyltransferase activity and are easier to identify targets [28]. It has been shown that P300 can induce GRP78 acetylation in breast cancer cells, so acetyltransferase P300 was selected in the present study [11]. Our study showed that P300 expression in rat myocardial tissue was increased markedly at 6 and 12 h after trauma, which was positively correlated with the level of GRP78 acetylation elevation. HDACs can be categorized into two main families: the first family mainly includes class I (HDAC1; 2; 3; 8), IIa (HDAC4; 5; 7; 9), IIb (HDAC6 and HDAC10), and VI (HDAC11); the second family mainly includes class III HDACs or Sirtuins (Sirt1-7) proteins [29]. HDAC6 plays its deacetylase role mainly in the cytoplasm to regulate the deacetylation of molecular chaperones [30]. As an important stress monitoring factor, HDAC6 can upregulate the expression of the molecular chaperone HSP90 when misfolded proteins are increased [ 16, 31, 32]. In the present study, we found that the expression of HDAC6 in myocardial tissue was obviously increased 3 h after trauma and then decreased gradually, suggesting that P300 and HDAC6 may jointly participate in GRP78 acetylation in myocardial tissue and that GRP78 acetylation is associated with an increase in P300 and a decrease in HDAC6.

In addition, elevation of HDAC6 is favorable for the clearance of misfolded protein aggregates [33], while reduction of HDAC6 promotes the occurrence of acetylation [34]. High-resolution mass spectrometry (HRMS) showed that many molecular chaperones are acetylated on multiple lysine residues, which may affect their chaperone function during the cellular stress response [ 31, 35]. Our present study demonstrated that no apoptosis occurred within 3 h after trauma even when HDAC6 expression was elevated. However, with the lapse of time and the gradual decrease in HDAC6, ERS-induced apoptosis of cardiomyocytes began to occur and peaked 12 h after trauma. During trauma-induced protracted ERS, although GRP78 expression remained at a high level, the acetylation level of GRP78 was increased, leading to cardiomyocyte apoptosis. Acetylated GRP78 may cause GRP78 to lose its molecular chaperone function. As a result, large amounts of misfolded proteins aggregate, finally causing cardiomyocyte apoptosis. In cases where no timely intervention can be performed to interfere with the primary etiology, it is primarily important to prevent protracted ERS-induced damage. The finding of the present study that increased GRP78 acetylation could cause cardiomyocyte apoptosis may provide an important intervention target.

TSA is an effective HDAC inhibitor and has entered clinical trials as an anti-cancer drug because it can arrest cancer cells at the G1 and G2 phases and induce cell differentiation [ 36‒ 38]. It was found in this study that TSA could increase the GRP78 acetylation and apoptosis levels of H9C2 cells cultured with rat serum and simultaneously decrease the protein expression of GRP78, suggesting that acetylation might lead to the reduction of functional GRP78 and then trigger the excessive activation of downstream signaling. Interestingly, the increase in GRP78 acetylation resulted in a decrease in GRP78 protein expression without a significant change in GRP78 mRNA expression. The possible reasons include three aspects: reduced translation levels can lead to decreased expression of GRP78 protein; enhanced function of proteolytic enzymes can induce increased degradation of GRP78 protein; GRP78 acetylation may reduce its stability and cause increased degradation. This result is consistent with the finding that HDAC6 reduction could induce a reduction in GRP78 expression [16]. However, the specific molecular mechanism by which acetylated GRP78 is involved in cardiomyocyte apoptosis in TISCI and how P300 and HDAC6 regulate GRP78 require further research.

**Figure 6:**
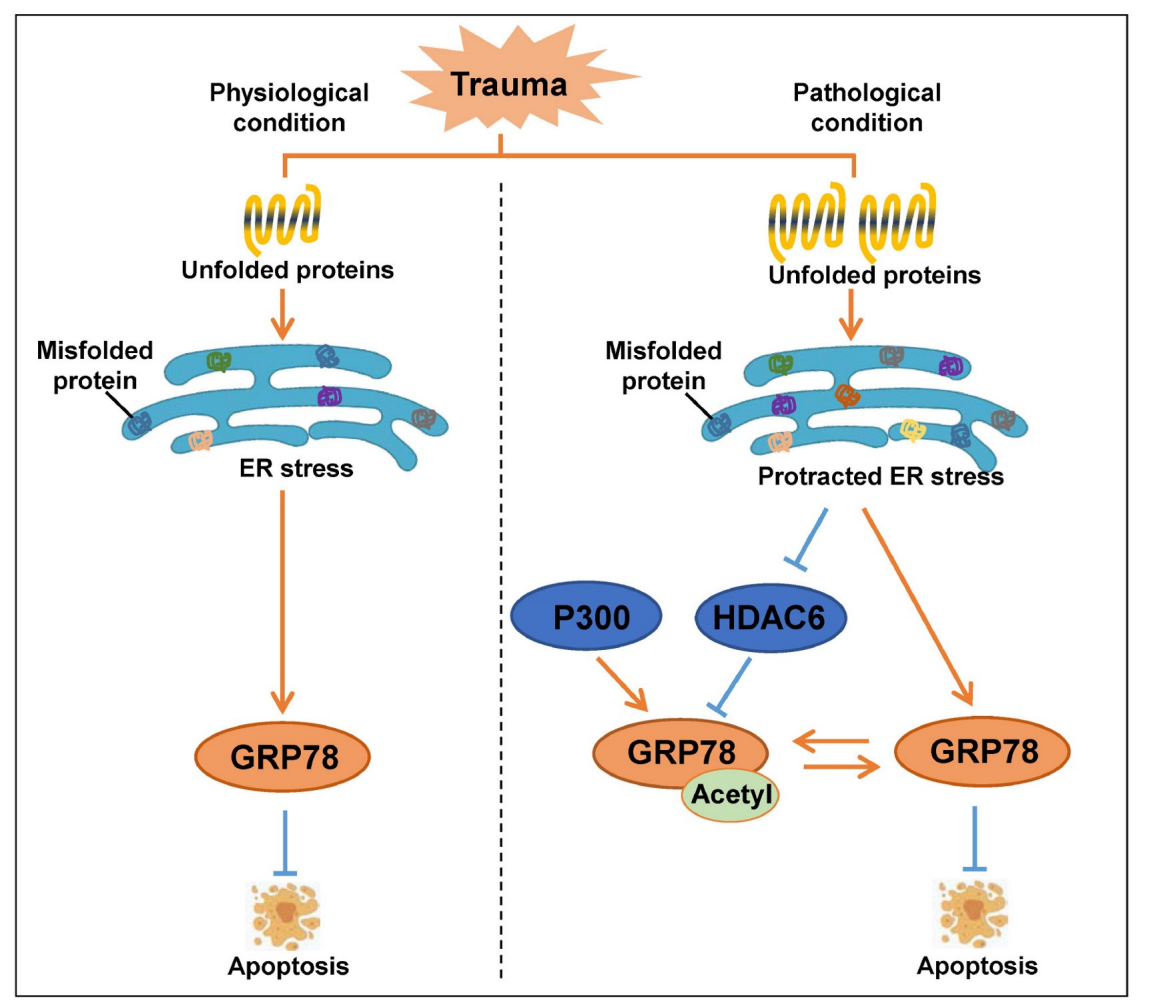
Figure 6 Schematic diagram of the mechanism by which GRP78 acetylation promotes apoptosis progression in cardiomyocytes Post-traumatic protracted ERS can promote cardiomyocyte apoptosis by increasing the acetylation level of GRP78.

In summary, with prolonged ERS after trauma, the increased acetylation level of GRP78 promotes cardiomyocyte apoptosis ( Figure 6). Reducing the acetylation level of GRP78 may reduce cell apoptosis and further improve cardiac function. These findings may provide an experimental basis for determining early molecular events in TISCI.
